# A Database of Dutch–English Cognates, Interlingual Homographs and Translation Equivalents

**DOI:** 10.5334/joc.67

**Published:** 2019-07-19

**Authors:** Eva D. Poort, Jennifer M. Rodd

**Affiliations:** 1 Department of Experimental Psychology, University College London, UK

**Keywords:** Bilingualism, Stimulus development, Word processing

## Abstract

To investigate the structure of the bilingual mental lexicon, researchers in the field of bilingualism often use words that exist in multiple languages: cognates (which have the same meaning) and interlingual homographs (which have a different meaning). A high proportion of these studies have investigated language processing in Dutch–English bilinguals. Despite the abundance of research using such materials, few studies exist that have validated such materials. We conducted two rating experiments in which Dutch–English bilinguals rated the meaning, spelling and pronunciation similarity of pairs of Dutch and English words. On the basis of these results, we present a new database of Dutch–English identical cognates (e.g. “wolf”–“wolf”; *n* = 58), non-identical cognates (e.g. “kat”–“cat”; *n* = 74), interlingual homographs (e.g. “angel”–“angel”; *n* = 72) and translation equivalents (e.g. “wortel”–“carrot”; *n* = 78). The database can be accessed at http://osf.io/tcdxb/.

## 1 Introduction

The study of the structure of the bilingual mental lexicon is a core area in the field of bilingualism. Researchers in this field often use words that exist in multiple languages to determine how the lexicon works in a bilingual. Most of this research to date has used cognates (e.g. Brenders, Van Hell, & Dijkstra, 2011; Bultena, Dijkstra, & Van Hell, 2014; Caramazza & Brones, 1979; Comesaña et al., 2015; Costa, Caramazza, & Sebastián-Gallés, 2000; Cristoffanini, Kirsner, & Milech, 1986; De Groot & Nas, 1991; Dijkstra, Grainger, & Van Heuven, 1999; Dijkstra, Miwa, Brummelhuis, Sappelli, & Baayen, 2010; Duyck, Van Assche, Drieghe, & Hartsuiker, 2007; Lemhöfer & Dijkstra, 2004; Lemhöfer, Dijkstra, & Michel, 2004; Libben & Titone, 2009; Peeters, Dijkstra, & Grainger, 2013; Poort & Rodd, 2017; Poort, Warren, & Rodd, 2016; Sánchez-Casas, García-Albea, & Davis, 1992; Schwartz & Kroll, 2006; Schwartz, Kroll, & Diaz, 2007; Titone, Libben, Mercier, Whitford, & Pivneva, 2011; Van Assche, Drieghe, Duyck, Welvaert, & Hartsuiker, 2011; Van Assche, Duyck, Hartsuiker, & Diependaele, 2009; Van Hell & De Groot, 1998, 2008; Van Hell & Dijkstra, 2002; Yudes, Macizo, & Bajo, 2010). Cognates are words that, in their purest definition, are spelled identically and have the same meaning in those languages: the Dutch word “wolf”, for example, means the same as the English word “wolf”. In addition to using identical cognates, many researchers also use non-identical cognates (e.g. Comesaña et al., 2015; Dijkstra et al., 2010; Duyck et al., 2007; Poort & Rodd, 2017, May 30; Van Assche et al., 2011), like the Dutch word “kat” and the English word “cat”. A substantial number of studies has also used identical interlingual homographs (e.g. De Bruijn, Dijkstra, Chwilla, & Schriefers, 2001; De Groot, Delmaar, & Lupker, 2000; Dijkstra, De Bruijn, Schriefers, & Ten Brinke, 2000; Dijkstra et al., 1999; Dijkstra, Van Jaarsveld, & Ten Brinke, 1998; Dijkstra, Timmermans, & Schriefers, 2000; Jared & Szucs, 2002; Kerkhofs, Dijkstra, Chwilla, & De Bruijn, 2006; Lemhöfer & Dijkstra, 2004; Libben & Titone, 2009; Macizo, Bajo, & Cruz Martín, 2010; Poort et al., 2016; Smits, Martensen, Dijkstra, & Sandra, 2006; Titone et al., 2011; Van Heuven, Schriefers, Dijkstra, & Hagoort, 2008; Von Studnitz & Green, 2002), which in counterpoint to cognates do not share their meaning across languages: the word “angel” in English refers to a heavenly being, while in Dutch it refers to the sting of a bee or wasp. These items are often compared to control words, or translation equivalents. Translation equivalents share their meaning but not their form: the Dutch word “wortel” is the translation equivalent of the English word “carrot”.

Despite the abundance of research using cognates and interlingual homographs, and the high proportion of those studies that investigated language processing in Dutch–English bilinguals specifically, few studies exist that have extensively validated or pre-tested such materials. Indeed, the biggest study to do so for Dutch and English that we are aware of was conducted by Dijkstra et al. (2010). As part of the stimulus development for a series of experiments examining the impact of cross-linguistic similarity, they asked Dutch–English bilinguals to rate pairs of Dutch and English words in terms of their semantic, orthographic and phonological similarity. This rating experiment yielded a set of 360 words, all of which had a semantic similarity rating of greater than 6 on their 7-point scale. Half of the items had an orthographic similarity rating of less than 2 (and were considered the control items in their subsequent experiments), while the other half had ratings greater than 2 (which were considered the ‘cognates’). Notably, however, only 31 items were identical cognates.

Another large study that collected similarity ratings from Dutch–English bilinguals was conducted by Tokowicz, Kroll, De Groot, and Van Hell (2002). Their aim was to collect number-of-translation norms for 562 Dutch–English translation pairs. As in the rating study conducted by Dijkstra et al. (2010), they asked Dutch–English bilinguals to rate the semantic similarity of these pairs of words but, in contrast to Dijkstra et al. (2010), their participants were asked to give a single ‘form’ similarity rating, taking both the pairs’ orthographic and phonological similarity into account. Again, the authors state that approximately 40% of these pairs could be considered cognates, but only 35 pairs were identical in form. In short, although resources exist that have validated the cross-linguistic similarity of pairs of Dutch and English words, these resources contain very few identical pairs and, if they do, most of these are cognates. No one has, as yet, attempted to validate a set of Dutch–English interlingual homographs.

The aim of the experiments presented here was to fill that gap. We conducted two rating experiments to develop a database of Dutch–English identical and non-identical cognates and identical interlingual homographs,^1^ as well as Dutch–English translation equivalents. The identical cognates, non-identical cognates and translation equivalents were rated in Experiment 1; the interlingual homographs were rated in Experiment 2.^2^ Similar to Dijkstra et al. (2010 and Tokowicz et al. (2002), we asked Dutch–English bilinguals to rate the items’ similarity in Dutch and English in terms of their meaning, spelling and pronunciation. Ratings were obtained for meaning, spelling and pronunciation similarity as these variables critically affect word processing in bilinguals (e.g. Dijkstra et al., 1999; Lemhöfer & Dijkstra, 2004). Phonological similarity is not usually considered a core feature of the definitions of the word types, however, so the pronunciation similarity ratings were obtained for the sake of completeness only and were not used to discard any items from the database. Furthermore, as we intended to use these items in a cross-lingual priming paradigm in which participants would first read Dutch sentences that contained one of the stimuli (see Poort and Rodd (2017, May 30) and Experiment 2 of Poort and Rodd (in press), we decided to provide these sentences in the rating experiments as well.

In these experiments, we adopted the following definitions of the critical word types:

- Identical cognates were defined as words that had an identical written form in both Dutch and English and highly similar meanings in both languages (e.g. “wolf”–“wolf”).
- Non-identical cognates were defined as having very similar but not identical forms in Dutch and English and highly similar meanings in both languages (e.g. “kat”–“cat”).
- Interlingual homographs were defined as having identical forms in Dutch and English, but different and unrelated meanings (e.g. “angel”–“angel”, where “angel” means “insect’s sting” in Dutch).
- Translation equivalents were defined as a pair of Dutch and English words that were translations of each other but whose written forms were not at all or only minimally similar (e.g. “wortel”–“carrot”).

## 2 Experiment 1

### 2.1 Methods

#### 2.1.1 Materials

We first collected an initial set of 103 identical cognates, all nouns and/or adjectives between 3 and 8 letters long. (Note that some of the items could also be used as verbs in English, such as “plan”–“plan”.) Sixty-one of these items were taken from Dijkstra et al. (2010) and Tokowicz et al. (2002). The rest of the identical cognates were selected from other published research articles (see Table 1). Of the 61 identical cognates selected from Dijkstra et al. (2010) and Tokowicz et al. (2002), we discarded all items with a meaning similarity rating that was less than 6 on their 7-point scales. We also discarded any items with a frequency in Dutch or English that was less than 2 occurrences per million according to the SUBTLEX-NL (Keuleers, Brysbaert, & New, 2010) and SUBTLEX-US (Brysbaert & New, 2009)^3^ databases and one item that had a mean lexical decision accuracy of less than 85% in the English Lexicon Project (Balota et al., 2007). Finally, we discarded any items that were only identical in form when inflected (e.g. “pure”–“pure”, where “pure” in Dutch is the inflected form of the adjective “puur”).

**Table 1:** Experiment 1 & 2. Published articles from which we selected many of the identical cognates and interlingual homographs that were rated in the two experiments. The first column lists the sources of identical cognates for the first experiment. The second column lists the sources of identical interlingual homographs for the second experiment.

Sources of identical cognates	Sources of identical interlingual homographs

Dijkstra, Grainger, and Van Heuven (1999)	Dijkstra, Grainger, and Van Heuven (1999)
Dijkstra, Van Jaarsveld, and Ten Brinke (1998)	Dijkstra, Timmermans, and Schriefers (2000)
Lemhöfer and Dijkstra (2004)	Dijkstra, Van Jaarsveld, and Ten Brinke (1998)
Peeters, Dijkstra, and Grainger (2013)	Kerkhofs, Dijkstra, Chwilla and De Bruijn (2006)
Poort, Warren, and Rodd (2016)	Poort, Warren, and Rodd (2016)
Van Hell and De Groot (1998)	Schulpen, Dijkstra, Schriefers, and Hasper (2003)
Van Hell and Dijkstra (2002)	Smits, Martensen, Dijkstra, and Sandra (2006)

Next, we collected an initial set of 134 non-identical cognates and 444 translation equivalents, again all nouns and/or adjectives between 3 and 8 letters long and with frequencies greater than 2 occurrences per million in both Dutch and English. We again selected only items that had received a meaning similarity rating greater than 6 on the 7-point scales used by Dijkstra et al. (2010) and Tokowicz et al. (2002). Furthermore, for the set of non-identical cognates, we selected only items with a score greater than 0.5 but less than 1 on an objective measure of orthographic overlap, which we calculated using the formula proposed by Schepens, Dijkstra, and Grootjens (2012): we divided the Levenshtein distance (Levenshtein, 1966) between the Dutch and English written forms of the word by the number of letters of the longest form of the word and subtracted this from 1. We also required that their form similarity rating (Tokowicz et al., 2002) or average orthography-phonology similarity rating (Dijkstra et al., 2010) was above 5. (Because Tokowicz et al. (2002) had asked their participants to take both spelling and pronunciation into account for a single ‘form similarity’ rating, we calculated an average of the orthographic and phonological similarity ratings items had received in the Dijkstra et al. (2010) study, to be more comparable to Tokowicz et al.’s (2002) form similarity rating.). The 444 translation equivalents had objective orthographic overlap scores of less than 0.5 and form similarity ratings (Tokowicz et al., 2002) or average of orthography-phonology similarity ratings (Dijkstra et al., 2010) of less than 3. All English forms of the items had a mean lexical decision accuracy in the English Lexicon Project (Balota et al., 2007) greater than 85%.

Because the identical cognates generally had lower frequency-of-use values than the non-identical cognates and translation equivalents, items with high frequency-of-use values were discarded. Similarly, the identical cognates were less orthographically complex than the non-identical cognates and translation equivalents, so items with a high OLD20 in either Dutch or English were excluded. A word’s OLD20 value is calculated as its mean orthographic Levenshtein distance to a its 20 closest neighbours (Yarkoni, Balota, & Yap, 2008). Finally, offensive words and items that could belong to more than one word type were excluded (e.g. the Dutch word “brood” is a non-identical cognate of the English word “bread”, but also an identical interlingual homograph of the English word “brood”).

After this second step in the selection procedure, a total of 65 identical cognates, 102 non-identical cognates and 315 translation equivalents remained. To determine the final set of stimuli to be rated, we let the software package Match (Van Casteren & Davis, 2007) select the 80 non-identical cognates and 80 translation equivalents that best matched the 65 identical cognates. Matching was based on log-transformed word frequency, word length and OLD20 in both Dutch and English. Note that, because Tokowicz et al.’s (2002) aim was to collect translation norms, many of the translation equivalents and some of the non-identical cognates appeared more than once in Tokowicz et al.’s (2002) materials with different translations (e.g. “afval”–“trash” and “afval”–“waste”). We manually made sure no word form was selected by Match twice. Table 2 lists means, minimums, maximums and standard deviations per word type for each of the matching measures (and raw word frequency) for both English and Dutch.

**Table 2:** Experiment 1 & 2. Means (and standard deviations) and minimum and maximum values for the Dutch and English characteristics and orthographic similarity measure for the 65 identical cognates, 80 non-identical cognates, 87 identical interlingual homographs and 80 translation equivalents rated across both experiments. Frequency refers to the word’s SUBTLEX frequency in occurrences per million [see Keuleers et al. (2010) for Dutch and Brysbaert & New (2009) for English]; log10(frequency) refers to the SUBTLEX log-transformed raw word frequency [log10(raw frequency+1)]; OLD20 refers to Yarkoni et al.’s (2008) measure of orthographic complexity of a word, expressed as its mean orthographic Levenshtein distance to its 20 closest neighbours; orthographic similarity refers to the measure of objective orthographic similarity discussed in the text (measured on a scale from 0 to 1), which was calculated as the Levenshtein distance between the Dutch and English forms of the words divided by the length of the longest of the two forms.

	Characteristics Dutch words				Characteristics English words				Orthographic similarity

	frequency	log10(frequency)	word length	OLD20	frequency	log10(frequency)	word length	OLD20	

identical cognates	41.5 (61.2) *min:* 2.17 *max:* 254	2.94 (0.51) *min:* 1.98 *max:* 4.05	4.52 (1.08) *min:* 3 *max:* 8	1.58 (0.42) *min:* 1.00 *max:* 2.50	44.9 (61.5) *min:* 2.35 *max:* 308	3.08 (0.49) *min:* 2.08 *max:* 4.20	4.52 (1.08) *min:* 3 *max:* 8	1.60 (0.36) *min:* 1.00 *max:* 2.60	1.00 (0.00) *min:* 1.00 *max:* 1.00
non-identical cognates	37.7 (44.7) *min:* 2.26 *max:* 244	2.95 (0.50) *min:* 2.00 *max:* 4.03	4.95 (1.05) *min:* 3 *max:* 8	1.55 (0.35) *min:* 1.00 *max:* 2.45	47.9 (57.0) *min:* 2.59 *max:* 266	3.15 (0.46) *min:* 2.12 *max:* 4.13	4.96 (1.00) *min:* 3 *max:* 8	1.69 (0.39) *min:* 1.00 *max:* 2.60	0.69 (0.12) *min:* 0.50 *max:* 0.83
interlingual homographs	39.2 (95.1) *min:* 0.09 *max:* 580	2.57 (0.77) *min:* 0.70 *max:* 4.40	4.22 (1.13) *min:* 3 *max:* 7	1.32 (0.37) *min:* 1.00 *max:* 2.70	65.8 (153) *min:* 0.22 *max:* 828	2.81 (0.81) *min:* 1.08 *max:* 4.63	4.22 (1.13) *min:* 3 *max:* 7	1.43 (0.36) *min:* 1.00 *max:* 2.80	1.00 (0.00) *min:* 1.00 *max:* 1.00
translation equivalents	34.1 (35.6) *min:* 2.15 *max:* 179	2.96 (0.45) *min:* 1.98 *max:* 3.89	4.90 (1.00) *min:* 3 *max:* 7	1.49 (0.31) *min:* 1.00 *max:* 2.25	37.5 (38.4) *min:* 3.63 *max:* 215	3.10 (0.41) *min:* 2.27 *max:* 4.04	4.64 (1.02) *min:* 3 *max:* 8	1.63 (0.34) *min:* 1.00 *max:* 2.50	0.11 (0.14) *min:* 0.00 *max:* 0.50

As mentioned in the Introduction, we intended to use these stimuli in a cross-lingual long-term priming experiment. In this experiment, the participants would first read Dutch sentences that contained one of the items. Therefore, the next step involved writing the Dutch sentences for the selected items (see Table 3 for example sentences). The sentences were between 6 and 12 words long and were written so that the target word was placed as far towards the end of the sentence as possible, as this minimises ambiguity. Each target word appeared only in its own sentence and not in any other sentence and only in its uninflected form (e.g. nouns were not pluralised). For nine of the non-identical cognates and 21 of the translation equivalents that Match selected, it was difficult to write a clear and concise sentence that complied with all of these criteria. These items were manually replaced with more suitable items of a similar frequency, length and OLD20.

**Table 3:** Experiment 1 & 2. Examples of items for each of the word types and the Dutch sentence that provided a context for the word (with English translations). The non-identical interlingual homographs only served as fillers in these experiments. The catch items were included to determine whether the participants were carefully reading the sentences. During the experiments, the participants were only shown the Dutch sentence (with the Dutch word form, as here, marked in bold) and the English word form.

	Dutch word form	English word form	Sentence (Dutch original)	Sentence (English translation)

identical cognate	wolf	wolf	De hond is een gedomesticeerde ondersoort van de **wolf**.	The dog is a domesticated subspecies of the **wolf**.
non-identical cognate	kat	cat	Haar ouders hebben een dikke, grijze **kat**.	Her parents have a fat, grey **cat**.
translation equivalent	wortel	carrot	Een ezel kun je altijd blij maken met een **wortel**.	You can always make a donkey happy with a **carrot**.
identical interlingual homograph	angel	angel	Alleen vrouwelijke bijen en wespen hebben een **angel**.	Only female bees and wasps have a **sting**.
non-identical interlingual homograph	brutaal	brutal	Als klein meisje was ze behoorlijk **brutaal**.	When she was a little girl she was quite **cheeky**.
catch item	vorst	frost	Een andere aanduiding voor monarch is **vorst**.	A different term for monarch is **sovereign**.

Finally, to ensure that the participants would make full use of the rating scale for all three aspects—meaning, spelling and pronunciation similarity—across all items, 40 identical interlingual homographs and 21 non-identical interlingual homographs were selected from Poort et al. (2016) and a list on Wikipedia (Wikipedia, 2014). We selected only words that were between 3 and 8 letters long. Any items for which either the Dutch or English frequency was less than 2 occurrences per million or more than 700 were discarded, as well as all items that belonged to more than one word type^4^ and items for which it was difficult to write a clear and concise Dutch sentence. This left 31 identical and 14 non-identical interlingual homograph pairs to serve as fillers in the first experiment. The sentences for these items were written according to the same criteria as for the identical and non-identical cognates and the translation equivalents. A native speaker of Dutch then proofread all 270 sentences for both the targets and fillers and suggested corrections and clarifications where necessary.

#### 2.1.2 Design and Procedure

The experiment was set up in Qualtrics (Qualtrics, 2015). Participants saw the English word (in bold) on the left and the Dutch sentence with the Dutch target word in bold on the right and were asked to rate, on a scale from 1 to 7, how similar the two words in bold were in terms of their meaning, spelling and pronunciation. As there were 225 target items, to reduce the total length of the experiment and minimise any effects of fatigue, we created five versions of the experiment, each containing 45 target items plus the 45 identical and non-identical interlingual homograph fillers. To allow us to check whether the participants had carefully read the sentences, each version also included an additional five catch trials for which the Dutch and English words could be translations of each other (varying in their degree of orthographic similarity), but in the context of the sentence the Dutch word required a different English translation. For example, the word “vorst” in Dutch can be translated as “frost” in English, but also as “monarch”. The word “vorst” was then used in a Dutch sentence to mean “monarch”, but the participants were asked to rate the similarity in meaning (and spelling and pronunciation) between “vorst” and “frost”. Participants were randomly assigned to one of the five versions of the experiment and the order of items was randomised individually for each participant. Only five items were presented per screen, for a total of 19 screens. At the start of the experiment, the participants were shown six examples (including an example of a catch item) with suggested ratings. They filled in a language background questionnaire at the end.

#### 2.1.3 Participants

Our aim was to recruit between 10 and 15 participants for each of the five versions of the experiment. Participants were eligible to participate in the experiment if they were a native speaker of Dutch and fluent speaker of English and had not been diagnosed with a language disorder. They also had to be between the ages of 18 and 50 and of Dutch or Belgian nationality. A total of 77 participants was recruited through personal contacts resident in the Netherlands, social media and word-of-mouth. The participants gave informed consent (by means of ticking a box on the online consent form) and participated for a chance to win an electronic gift card worth €100 (then roughly £75). The UCL Experimental Psychology Ethics Committee provided approval of our study protocol (Project ID: fMRI/2013/016).

The data from one participant were excluded because this participant regularly rated the spelling and pronunciation similarity of the identical and non-identical cognates a 1 or 2. The data from an additional nine participants were excluded because these participants made more than three mistakes on the five catch trials.

The remaining 67 participants (14 males; *M*_age_ = 23.5 years, *SD*_age_ = 5.4 years) had started learning English from an average age of 7.7 (*SD* = 3.3 years) and so had an average of 15.8 years of experience with English (*SD* = 5.8 years). The participants rated their proficiency as 9.7 out of 10 in Dutch (*SD* = 0.6) and 9.2 in English (*SD* = 0.7). A two-sided paired *t*-test showed this difference to be significant [*t*(66) = 4.729, *p* < .001]. The five versions were completed by 13, 14, 12, 15 and 13 participants respectively. There were no differences between the versions with respect to the demographic variables reported here (as shown by ANOVAs and chi-square tests where appropriate; all *p*s > .125).

### 2.2 Findings

Mean ratings for the three word types (identical cognates, non-identical cognates and translation equivalents) for all three aspects (meaning, spelling and pronunciation similarity) can be found in Table 4. Overall, most items had received high (or low) ratings for the three aspects as expected for their word type.

**Table 4:** Experiment 1 & 2. Means (and standard deviations) and minimum and maximum values for the Dutch and English characteristics and similarity ratings for the set 58 identical cognates, 76 non-identical cognates, 72 identical interlingual homographs and 78 translation equivalents selected for inclusion in our database. Frequency refers to the word’s SUBTLEX frequency in occurrences per million [see Keuleers et al. (2010) for Dutch and Brysbaert & New (2009) for English]; log10(frequency) refers to the SUBTLEX log-transformed raw word frequency [log10(raw frequency+1)]; OLD20 refers to Yarkoni et al.’s (2008) measure of orthographic complexity of a word, expressed as its mean orthographic Levenshtein distance to its 20 closest neighbours. The similarity ratings were provided on a scale from 1 (not at all similar) to 7 [(almost) identical]. For the 28 items (7 identical cognates, 7 non-identical cognates and 14 translation equivalents) that were included in both experiments, only the average ratings from the first experiment were used.

	Characteristics Dutch words				Characteristics English words				Similarity ratings		

	frequency	log10(frequency)	word length	OLD20	frequency	log10(frequency)	word length	OLD20	meaning	spelling	pronunciation

identical cognates	37.0 (56.3) *min:* 2.17 *max:* 254	2.90 (0.49) *min:* 1.98 *max:* 4.05	4.57 (1.11) *min:* 3 *max:* 8	1.61 (0.42) *min:* 1.00 *max:* 2.50	41.5 (54.0) *min:* 2.35 *max:* 280	3.07 (0.47) *min:* 2.08 *max:* 4.15	4.57 (1.11) *min:* 3 *max:* 8	1.63 (0.35) *min:* 1.00 *max:* 2.60	6.83 (0.22) *min:* 6.20 *max:* 7.00	7.00 (0.02) *min:* 6.92 *max:* 7.00	5.91 (0.67) *min:* 4.21 *max:* 7.00
non-identical cognates	38.3 (45.6) *min:* 2.26 *max:* 244	2.96 (0.50) *min:* 2.00 *max:* 4.03	5.00 (1.06) *min:* 3 *max:* 8	1.57 (0.35) *min:* 1.00 *max:* 2.45	48.8 (58.1) *min:* 2.59 *max:* 266	3.16 (0.46) *min:* 2.12 *max:* 4.13	4.99 (1.01) *min:* 3 *max:* 8	1.69 (0.39) *min:* 1.00 *max:* 2.55	6.86 (0.21) *min:* 6.00 *max:* 7.00	5.35 (0.53) *min:* 4.00 *max:* 6.08	5.06 (0.72) *min:* 3.62 *max:* 6.80
interlingual homographs	55.4 (126) *min:* 0.09 *max:* 662	2.74 (0.74) *min:* 0.70 *max:* 4.46	3.96 (0.86) *min:* 3 *max:* 7	1.26 (0.32) *min:* 1.00 *max:* 2.70	70.9 (163) *min:* 0.29 *max:* 828	2.91 (0.73) *min:* 1.20 *max:* 4.63	4.01 (0.94) *min:* 3 *max:* 7	1.37 (0.32) *min:* 1.00 *max:* 2.80	1.16 (0.28) *min:* 1.00 *max:* 2.20	7.00 (0.01) *min:* 6.91 *max:* 7.00	5.49 (0.79) *min:* 3.83 *max:* 7.00
translation equivalents	33.5 (35.2) *min:* 2.15 *max:* 179	2.95 (0.45) *min:* 1.98 *max:* 3.89	4.90 (1.00) *min:* 3 *max:* 7	1.49 (0.31) *min:* 1.00 *max:* 2.25	35.4 (33.1) *min:* 3.63 *max:* 175	3.09 (0.40) *min:* 2.27 *max:* 3.95	4.63 (1.02) *min:* 3 *max:* 8	1.63 (0.33) *min:* 1.00 *max:* 2.50	6.88 (0.17) *min:* 6.23 *max:* 7.00	1.20 (0.43) *min:* 1.00 *max:* 2.92	1.18 (0.41) *min:* 1.00 *max:* 3.08

All translation equivalents received meaning similarity ratings of 6 or greater. Seven identical and three non-identical cognates with meaning similarity ratings below 6 on the 7-point scale were discarded from the database. Unexpectedly, two identical cognates (“crisis”–“crisis” and “lens”–“lens”) received spelling similarity ratings of less than 7. Since these two items were truly identical, they were not discarded. Two translation equivalents with spelling similarity ratings higher than 3 were discarded. Our intention was also to discard all non-identical cognates with spelling similarity ratings of less than 5, in line with the initial selection criteria, but 21 non-identical cognates met this criterion. In order not to reduce the number of stimuli too much, only the one non-identical cognate with a spelling similarity rating of less than 4 was discarded. In conclusion, the first experiment produced a database of stimuli that included 58 identical cognates, 76 non-identical cognates and 78 translation equivalents.

## 3 Experiment 2

A second experiment was conducted to produce a database of identical interlingual homographs. This second experiment was designed in an identical manner as the first experiment.

### 3.1 Methods

#### 3.1.1 Materials

Seventy additional identical interlingual homographs between 3 and 8 letters long were selected from the research articles listed in Table 1 or a list of identical entries in the SUBTLEX-US and SUBTLEX-NL databases (Brysbaert & New, 2009; Keuleers et al., 2010, respectively). In the latter case, all noun, verb and adjective entries between 3 and 8 letters long were extracted from the SUBTLEX-US and SUBTLEX-NL databases and those with identical forms but dissimilar meanings in Dutch and English (as judged by the first author) were manually selected.

As in Experiment 1, from this initial selection any items that had a mean lexical decision accuracy in the English Lexicon Project (Balota et al., 2007) of less than 85% were discarded. Since it was more difficult to find identical interlingual homographs, items with frequencies of less than 2 occurrences per million that were considered to be well-known words regardless were retained, as well as words with a very high frequency or high OLD20. Similarly, we also included items that could be a (non-)identical cognate (e.g. “lever”–“lever”, where “lever” in Dutch is also a non-identical cognate with the English word “liver”). Lastly, items for which it was difficult to write a clear and concise sentence in Dutch were excluded, as well as items that were only identical when inflected. A total of 56 items met these criteria. Table 2 lists means, minimums, maximums and standard deviations for each of these measures (and raw word frequency) for both English and Dutch. The sentences for these items were written according to the same criteria as for the first experiment and were proofread by the same native speaker of Dutch who proofread those sentences. Finally, to ensure again that the participants would make full use of the entire rating scale across all items for all three aspects they were asked to judge, seven identical cognates, seven non-identical cognates, seven non-identical interlingual homographs and 14 translation equivalents were selected from the materials for the first experiment to serve as fillers in the second experiment.

#### 3.1.2 Design and Procedure

The experimental design and procedure of the second experiment was the same as that of the first, except that participants were now also able to indicate if they were not familiar with a word, as not all words met the desired frequency criteria. Two versions of the experiment were created, each containing 28 targets plus the 35 identical and non-identical cognate and translation equivalent fillers and the five catch trials from the first experiment.

#### 3.1.3 Participants

Again, our aim was to recruit between 10 and 15 participants for each of the two versions of the experiment. A total of 24 participants was recruited using the same eligibility criteria and recruitment procedure as for the first experiment. The participants again gave informed consent (by means of ticking a box on the online consent form) and participated for a chance to win an electronic gift card worth €75 (then roughly £55). The UCL Experimental Psychology Ethics Committee provided approval of our study protocol (Project ID: fMRI/2013/016).

The data from one participant were excluded because this participant regularly rated the spelling and pronunciation similarity of the identical and non-identical cognates a 1 or 2. No participants made more than three mistakes on the five catch trials.

The remaining 23 participants (8 males; *M*_age_ = 24.5 years, *SD*_age_ = 5.9 years) had started learning English from an average age of 6.3 (*SD* = 4.0 years) and so had an average of 18.2 years of experience with English (*SD* = 5.0 years). The participants rated their proficiency as 9.5 out of 10 in Dutch (*SD* = 0.7) and 9.2 in English (*SD* = 0.7). A two-sided paired *t*-test showed this difference to be non-significant [*t*(66) = 1.628, *p* = .118]. Eleven participants completed version 1 and 12 completed version 2. A two-sided independent-samples Welch’s *t*-test showed that there was a significant difference in age between the two versions [version 1: *M* = 22.4 years, *SD* = 1.9 years; version 2: *M* = 26.5 years, *SD* = 6.0 years; *t*(13.4) = 2.264, *p* = .041]. There were no significant differences between the versions with respect to the other demographic variables reported here (as shown by additional independent-samples Welch’s *t*-tests and chi-square tests where appropriate; all *p*s > .1).

### 3.2 Findings

Mean ratings for the identical interlingual homographs for all three aspects (meaning, spelling and pronunciation similarity) can be found in Table 4. Of the 87 interlingual homographs that had been rated in total across both experiments, most had received high (or low) ratings as expected for the three aspects. Again, five items (“angel”–“angel”, “fee”–“fee”, “peer”–“peer”, “steel”–“steel” and “wand”–“wand”) had strangely received spelling similarity ratings of less than 7, but these were retained as they were truly identical.

A total of 15 identical interlingual homographs was discarded from the database. One item in retrospect should not have been included in the pre-test because it had a mean accuracy in the English Lexicon Project (Balota et al., 2007) of less than 85%. Twelve other items were discarded because they had received an average meaning similarity rating greater than 2. Three other items (“honk”–“honk”, “lever”–“lever” and “stadium”–“stadium”) had also received an average meaning similarity rating greater than 2, but this was due to one or two participants giving them a high rating of 7 while all other participants had given them a rating of 1 or 2. As the majority of participants agreed that these items’ meanings were highly dissimilar, they were retained. Finally, two of the items that had been included in the second experiment were discarded because they had received ratings from fewer than ten participants, as some participants had indicated that they did not know those items. In total, the first and second experiment combined yielded a set of 72 identical interlingual homographs to add to the database.

#### 3.2.1 Additional analyses

##### 3.2.1.1 Between-experiment comparisons

To determine whether participants in the second experiment used the rating scales in a consistently different manner, we compared the ratings from the two experiments for the 28 identical cognates, non-identical cognates and translation equivalents that had been included in the first experiment as targets and in the second experiment as fillers. Overall, the differences between the ratings from the two experiments for the three properties were small. (Positive differences indicate higher ratings were given in Experiment 2.) For meaning similarity, the average difference was 0.04 (*SD* = 0.16, *range* = –0.43–0.36). For spelling similarity, it was –0.04 (*SD* = 0.17, *range* = –0.34–0.61). Finally, for pronunciation similarity, the average difference was –0.01 (*SD* = 0.16, *range* = –0.25–0.50). Two-tailed one-sample *t*-tests indicated that these differences between the two experiments were not significant for any of the three properties [for meaning similarity: *t*(27) = 1.495, *p* = .147; for spelling similarity: *t*(27) = 1.379, *p* = .179; for pronunciation similarity: *t*(27) = 0.489, *p* = .629].

##### 3.2.1.2 Correlation analyses

**Figure 1:**
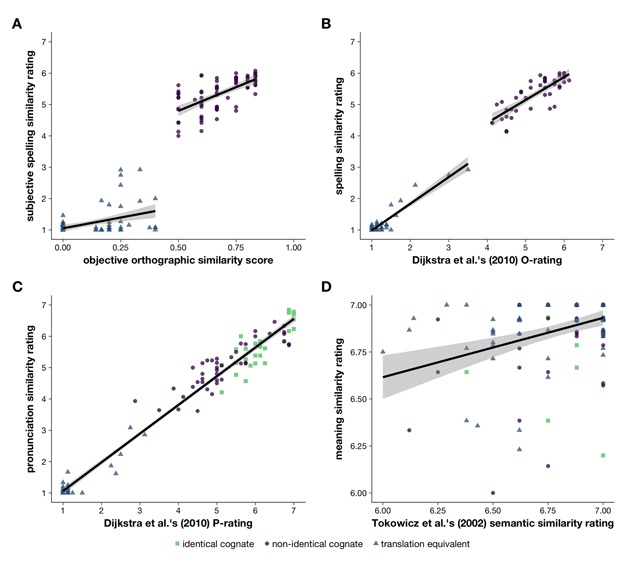
**A** Objective orthographic similarity score (x-axis) plotted against subjective spelling similarity rating (y-axis). **B** Dijkstra et al.’s (2010) orthographic similarity rating (O-rating; x-axis) plotted against the spelling similarity ratings obtained in the current experiments (y-axis). **C** Dijkstra et al.’s (2010) phonological similarity rating (P-rating; x-axis) plotted against the pronunciation similarity ratings obtained in the current experiments (y-axis). **D** Tokowicz et al.’s (2002) semantic similarity rating (x-axis) plotted against the meaning similarity ratings obtained in the current experiments (y-axis). Panels A and B display two regression lines fitted separately for each word type, while panels C and D display a single regression line fitted across all items. Word types are distinguished by colours and shapes (identical cognates, squares in green; non-identical cognates, circles in purple; translation equivalents, triangles in blue).

We computed correlations to assess the relationship between the objective orthographic similarity scores calculated using Schepens et al.’s (2012) formula, which we used to select our items, and the subjective spelling similarity ratings the items received in the experiments. We only included the non-identical cognates and the translation equivalents in these analyses, as the identical cognates and interlingual homographs by design all had a score of 1 on the objective orthographic similarity measure and (nearly all) had received a mean subjective spelling similarity rating of 7. We computed separate correlations for the non-identical cognates and the translation equivalents, as the non-identical cognates had been chosen because they had high objective orthographic similarity scores and the translation equivalents had been chosen because they had low scores. The scatterplots in panel A of Figure 1 demonstrate this discontinuity. For the non-identical cognates, the correlation between the objective orthographic similarity scores and the subjective spelling similarity ratings was strong and significant [*r*(74) = .657; 95% CI: .506–.768, *p* < .001]. For the translation equivalents, the correlation was somewhat less strong but still significant [*r*(76) = .417; 95% CI: .214–.585, *p* < .001].

We also computed correlations to determine whether our ratings were similar to those obtained by Dijkstra et al. (2010) and Tokowicz et al. (2002). Note that these analyses did not include the identical interlingual homographs, as these items had not been included in either of these two studies. First, we computed correlations for the relationship between Dijkstra et al.’s (2010) orthographic similarity ratings and the spelling similarity ratings we had obtained. These analyses also did not include the identical cognates, as they had nearly all received mean ratings of 7 in both our experiments and Dijkstra et al.’s (2010) experiment. As for the correlation between the objective orthographic similarity scores and subjective spelling similarity ratings, the scatterplot in panel B of Figure 1 showed a discontinuity in the data, so we computed separate correlations for the non-identical cognates and translation equivalents. For the translation equivalents, the correlation was near-perfect and significant [*r*(42) = .938; 95% CI: .889–.966, *p* < .001]. For the non-identical cognates, the correlation was similarly highly significant but slightly less strong [*r*(41) = .810; 95% CI: .674–.893, *p* < .001].

Second, we computed correlations for the relationship between Dijkstra et al.’s (2010) phonological similarity ratings and the pronunciation similarity ratings we had obtained. These analyses did include the identical cognates. As there was no discontinuity between the word types, nor did the scatterplot in panel C of Figure 1 suggest that the strength of the relationship between these two variables differed between the word types, we computed this correlation only across all items. The correlation was near-perfect and highly significant [*r*(111) = .985; 95% CI: .979–.990, *p* < .001].

Finally, we computed correlations for the relationship between Tokowicz et al.’s (2002) semantic similarity ratings and the meaning similarity ratings we had obtained in these experiments. Again, we computed this correlation only across all items, as the scatterplot in panel D of Figure 1 did not show a discontinuity between the word types, nor did it suggest that the strength of the relationship between these two variables differed between the word types. This correlation was of medium size but significant [*r*(134) = .365; 95% CI: .209–.502, *p* < .001].

## 4 Discussion

The two experiments presented in this paper have produced a database of experimentally validated Dutch–English identical cognates (*n* = 58), non-identical cognates (*n* = 76), identical interlingual homographs (*n* = 72) and translation equivalents (*n* = 78). While all of the non-identical cognates and translation equivalents had previously been validated in similar experiments (Dijkstra et al., 2010; Tokowicz et al., 2002), in contrast to this previous work, we also validated a large set of identical cognates and interlingual homographs. Our items were rated in two rating experiments, where we asked participants to rate the items’ spelling, pronunciation and meaning similarity in Dutch and English. One-sample *t*-tests showed that the ratings did not differ significantly between the two experiments. This indicates that there was no significant, consistent shift in how participants used the three scales in the two experiments. Furthermore, the spelling and pronunciation similarity ratings we obtained for the subset of items that had been included in Dijkstra et al.’s (2010) study correlated near-perfectly with the ratings obtained by Dijkstra et al. (2010) themselves. This provides further evidence of the validity of our ratings.

The correlation between our meaning similarity ratings and Tokowicz et al.’s (2002) semantic similarity ratings was considerably less strong (.365). As has been noted frequently, however, when either or both of the two variables involved in a bivariate correlation is restricted in range, this often leads to an underestimate of the correlation in the sample compared to the true correlation in the population (e.g. Alexander, Barrett, Alliger, & Carson, 1986; Bobko, 1983; Sackett & Yang, 2000; Thorndike, 1949). In our case, we had specifically selected items from Tokowicz et al.’s (2002) materials that had semantic similarity ratings greater than 6, effectively restricting both the range of their ratings and our own. Had we obtained similarity ratings for the full set of items included by Tokowicz et al. (2002), it is likely that our meaning similarity ratings would have correlated more strongly with Tokowicz et al.’s (2002) semantic similarity ratings.

The correlations between the objective orthographic similarity scores calculated using the formula proposed by Schepens et al. (2012) and the subjective spelling similarity ratings for the non-identical cognates and translation equivalents were of medium to strong size. This suggests that the spelling similarity ratings were influenced by other factors than merely the orthographic similarity of the items, such as by the pronunciation similarity of the items or cross-lingual spelling regularities. Notably, Schepens et al. (2012) themselves report a correlation of .96 with Dijkstra et al.’s (2010) orthographic similarity ratings. Most likely our correlations were lower because the non-identical cognates and translation equivalents had been dichotomised both with respect to the objective orthographic similarity scores and the subjective spelling similarity ratings. Consequently, only relatively few items had scores around the 0.50 mark and/or ratings in the 2–5 range. In contrast, Schepens et al. (2012) had computed their correlations across the full range of objective orthographic similarity scores and subjective spelling similarity ratings.

Researchers interested in using these stimuli should note we provided the participants with a sentence in Dutch for each item, as we intended to use these items in a series of cross-lingual long-term priming experiment. This may also have contributed to the low correlation we observed between our meaning similarity ratings and Tokowicz et al.’s (2002) semantic similarity ratings. Many of the non-identical cognates and translation equivalents had multiple meanings or senses in Dutch or English (or even multiple translations) and our sentences were, of course, confined to using only of those. In contrast, the participants in Tokowicz et al.’s (2002) study were free to think of whichever meaning(s) or sense(s) of these items they could think of, which likely resulted in differences in how their participants rated the meaning similarity of those items compared to our participants. However, the aforementioned effect of restricting the range of the ratings makes it difficult to say whether the observed correlation was low because we provided a sentence context for the Dutch word forms. Lastly, researchers should also note that the items in our database (especially the interlingual homographs) often have a different grammatical class in Dutch and English. Because many of these items are often also syntactically ambiguous within Dutch and/or English, we have not matched or labelled the items with respect to grammatical class.

To conclude, these stimuli will be useful for future research into the structure of the bilingual mental lexicon and bilingual language processing in general. While we intended to use these stimuli in experiments focusing on visual word processing using lexical decision tasks, we encourage researchers to use a range of various types of tasks and paradigms to further explore the differences we have observed between using a lexical decision task and a semantic relatedness judgement task (see Poort & Rodd, in press). Furthermore, we do not believe that providing a sentence context in the rating experiments affects whether these items can be used in isolation in future experiments, as we have used a subset of these items to successfully replicate both the cognate facilitation effect and the interlingual homograph inhibition effect (see Poort & Rodd, 2017). Nevertheless, researchers should keep in mind that many of the items we have included in our database have more than one meaning. Finally, the presence of the pronunciation similarity ratings also makes these stimuli suitable to further investigate the role that phonological similarity plays in the processing of cognates and interlingual homographs, which while important has received little attention (but see Dijkstra et al., 1999; Lemhöfer & Dijkstra, 2004). As such, this is an extremely valuable resource for researchers studying language processing in Dutch–English bilinguals.

## Data Accessibility Statement

The database can be accessed at http://osf.io/tcdxb/. An R script that contains the reported analyses can be accessed through the same link. Stimuli from this database have been used in experiments conducted by Poort and Rodd (2017), Poort and Rodd (2017, May 30) and Poort and Rodd (2019).

## Footnotes

Non-identical interlingual homographs technically also exist (e.g. the Dutch word “prei” means “leek” while the English word “prey” refers to something that is hunted), but these are much harder to operationalise. This is most likely because few bilinguals would consider the ‘conflict’ for a non-identical interlingual homograph to be as strong as for an identical interlingual homograph. Consequently, these items are not often used in research and we did not set out to validate a set of non-identical interlingual homographs.

The two experiments were conducted separately for the simple reason that we initially set out only to create a database of identical and non-identical cognates and translation equivalents. However, as the Additional analyses reported for the second experiment show, the participants in the two experiments did not use the scales in a meaningfully different manner. This indicates that the fact that the ratings were obtained in separate experiments does not affect their validity.

The SUBTLEX-US database (Brysbaert & New, 2009) was used instead of the SUBTLEX-UK database (Van Heuven, Mandera, Keuleers, & Brysbaert, 2014) because a regression analysis of data collected by Poort et al. (2016) revealed that the US frequencies were a better predictor of their Dutch–English bilinguals’ lexical decision times than the UK frequencies. Also note that the accuracy of the frequency measures of both databases is roughly equivalent, as the SUBTLEX-NL database (Keuleers et al., 2010) counted 43, 729, 424 tokens and the SUBTLEX-US database (Brysbaert & New, 2009) counted 51.0 million tokens.

A few of these items slipped the net, like “beer”–“beer”. (The identical interlingual homograph “beer” if used in Dutch would translate to “bear” in English and so is also a non-identical cognate; furthermore, when used in English, “beer” would translate to “bier” in Dutch).

## References

[1] Alexander, R. A., Barrett, G. V., Alliger, G. M., & Carson, K. P. (1986). Towards a general model of non-random sampling and the impact on population correlation: Generalizations of Berkson’s Fallacy and restriction of range. British Journal of Mathematical and Statistical Psychology, 39(1), 90–105. DOI: 10.1111/j.2044-8317.1986.tb00849.x 3768267

[2] Balota, D. A., Yap, M. J., Hutchison, K. A., Cortese, M. J., Kessler, B., Loftis, B., Treiman, R., et al. (2007). The English Lexicon Project. Behavior Research Methods, 39(3), 445–459. DOI: 10.3758/BF03193014 17958156

[3] Bobko, P. (1983). An analysis of correlations corrected for attenuation and range restriction. Journal of Applied Psychology, 68(4), 584–589. DOI: 10.1037/0021-9010.68.4.584

[4] Brenders, P., Van Hell, J. G., & Dijkstra, T. (2011). Word recognition in child second language learners: Evidence from cognates and false friends. Journal of Experimental Child Psychology, 109(4), 383–396. DOI: 10.1016/j.jecp.2011.03.012 21507422

[5] Brysbaert, M., & New, B. (2009). Moving beyond Kučera and Francis: A critical evaluation of current word frequency norms and the introduction of a new and improved word frequency measure for American English. Behavior Research Methods, 41(4), 977–990. DOI: 10.3758/BRM.41.4.977 19897807

[6] Bultena, S., Dijkstra, T., & Van Hell, J. G. (2014). Cognate effects in sentence context depend on word class, L2 proficiency, and task. Quarterly Journal of Experimental Psychology, 67(6), 1214–1241. DOI: 10.1080/17470218.2013.853090 24295513

[7] Caramazza, A., & Brones, I. (1979). Lexical access in bilinguals. Bulletin of the Psychonomic Society, 13(4), 212–214. DOI: 10.3758/BF03335062

[8] Comesaña, M., Ferré, P., Romero, J., Guasch, M., Soares, A. P., & García-Chico, T. (2015). Facilitative effect of cognate words vanishes when reducing the orthographic overlap: The role of stimuli list composition. Journal of Experimental Psychology: Learning, Memory, and Cognition, 41(3), 614–635. DOI: 10.1037/xlm0000065 25329085

[9] Costa, A., Caramazza, A., & Sebastián-Gallés, N. (2000). The cognate facilitation effect: Implications for models of lexical access. Journal of Experimental Psychology: Learning, Memory, and Cognition, 26(5), 1283–1296. DOI: 10.1037/0278-7393.26.5.1283 11009258

[10] Cristoffanini, P., Kirsner, K., & Milech, D. (1986). Bilingual lexical representation: The status of Spanish–English cognates. The Quarterly Journal of Experimental Psychology, 38(3), 367–393. DOI: 10.1080/14640748608401604

[11] De Bruijn, E. R. A., Dijkstra, T., Chwilla, D. J., & Schriefers, H. J. (2001). Language context effects on interlingual homograph recognition: Evidence from event-related potentials and response times in semantic priming. Bilingualism: Language and Cognition, 4(2), 155–168. DOI: 10.1017/S1366728901000256

[12] De Groot, A. M. B., Delmaar, P., & Lupker, S. J. (2000). The processing of interlexical homographs in translation recognition and lexical decision: Support for non-selective access to bilingual memory. The Quarterly Journal of Experimental Psychology: Section A, 53(2), 397–428. DOI: 10.1080/713755891 10881612

[13] De Groot, A. M. B., & Nas, G. L. J. (1991). Lexical representation of cognates and noncognates in compound bilinguals. Journal of Memory and Language, 30(1), 90–123. DOI: 10.1016/0749-596X(91)90012-9

[14] Dijkstra, T., De Bruijn, E., Schriefers, H., & Ten Brinke, S. (2000). More on interlingual homograph recognition: Language intermixing versus explicitness of instruction. Bilingualism: Language and Cognition, 3(1), 69–78. DOI: 10.1017/S1366728900000146

[15] Dijkstra, T., Grainger, J., & Van Heuven, W. J. B. (1999). Recognition of cognates and interlingual homographs: The neglected role of phonology. Journal of Memory and Language, 41(4), 496–518. DOI: 10.1006/jmla.1999.2654

[16] Dijkstra, T., Miwa, K., Brummelhuis, B., Sappelli, M., & Baayen, R. H. (2010). How cross-language similarity and task demands affect cognate recognition. Journal of Memory and Language, 62(3), 284–301. DOI: 10.1016/j.jml.2009.12.003

[17] Dijkstra, T., Timmermans, M., & Schriefers, H. J. (2000). On being blinded by your other language: Effects of task demands on interlingual homograph recognition. Journal of Memory and Language, 42(4), 445–464. DOI: 10.1006/jmla.1999.2697

[18] Dijkstra, T., Van Jaarsveld, H., & Ten Brinke, S. (1998). Interlingual homograph recognition: Effects of task demands and language intermixing. Bilingualism: Language and Cognition, 1(1), 51–66. DOI: 10.1017/S1366728998000121

[19] Duyck, W., Van Assche, E., Drieghe, D., & Hartsuiker, R. J. (2007). Visual word recognition by bilinguals in a sentence context: evidence for nonselective lexical access. Journal of Experimental Psychology: Learning, Memory, and Cognition, 33(4), 663–679. DOI: 10.1037/0278-7393.33.4.663 17576146

[20] Jared, D., & Szucs, C. (2002). Phonological activation in bilinguals: Evidence from interlingual homograph naming. Bilingualism: Language and Cognition, 5(3), 225–239. DOI: 10.1017/S1366728902003024

[21] Kerkhofs, R., Dijkstra, T., Chwilla, D. J., & De Bruijn, E. R. A. (2006). Testing a model for bilingual semantic priming with interlingual homographs: RT and N400 effects. Brain Research, 1068(1), 170–183. DOI: 10.1016/j.brainres.2005.10.087 16375868

[22] Keuleers, E., Brysbaert, M., & New, B. (2010). SUBTLEX-NL: A new measure for Dutch word frequency based on film subtitles. Behavior Research Methods, 42(3), 643–650. DOI: 10.3758/BRM.42.3.643 20805586

[23] Lemhöfer, K., & Dijkstra, T. (2004). Recognizing cognates and interlingual homographs: Effects of code similarity in language-specific and generalized lexical decision. Memory & Cognition, 32(4), 533–550. DOI: 10.3758/BF03195845 15478748

[24] Lemhöfer, K., Dijkstra, T., & Michel, M. (2004). Three languages, one ECHO: Cognate effects in trilingual word recognition. Language and Cognitive Processes, 19(5), 585–611. DOI: 10.1080/01690960444000007

[25] Levenshtein, V. I. (1966). Binary codes capable of correcting deletions, insertions, and reversals. Soviet Physics-Doklady, 10(8), 707–710.

[26] Libben, M. R., & Titone, D. A. (2009). Bilingual lexical access in context: Evidence from eye movements during reading. Journal of Experimental Psychology: Learning, Memory, and Cognition, 35(2), 381–390. DOI: 10.1037/a0014875 19271853

[27] Macizo, P., Bajo, T., & Cruz Martín, M. (2010). Inhibitory processes in bilingual language comprehension: Evidence from Spanish–English interlexical homographs. Journal of Memory and Language, 63(2), 232–244. DOI: 10.1016/j.jml.2010.04.002

[28] Peeters, D., Dijkstra, T., & Grainger, J. (2013). The representation and processing of identical cognates by late bilinguals: RT and ERP effects. Journal of Memory and Language, 68(4), 315–332. DOI: 10.1016/j.jml.2012.12.003

[29] Poort, E. D., & Rodd, J. M. (2017, 5 30). Studies of cross-lingual long-term priming. PsyArXiv. DOI: 10.31234/osf.io/ert8k

[30] Poort, E. D., & Rodd, J. M. (2017). The cognate facilitation effect in bilingual lexical decision is influenced by stimulus list composition. Acta Psychologica, 180, 52–63. DOI: 10.1016/j.actpsy.2017.08.008 28869840

[31] Poort, E. D., & Rodd, J. M. (2019). Towards a distributed connectionist account of cognates and interlingual homographs: Evidence from semantic relatedness tasks. PeerJ, 7: e6725 DOI: 10.7717/peerj.6725 31143528 PMC6526012

[32] Poort, E. D., Warren, J. E., & Rodd, J. M. (2016). Recent experience with cognates and interlingual homographs in one language affects subsequent processing in another language. Bilingualism: Language and Cognition, 19(1), 206–212. DOI: 10.1017/S1366728915000395

[33] Qualtrics. (2015). Qualtrics Survey Software. Provo, Utah, USA: Qualtrics.

[34] Sackett, P. R., & Yang, H. (2000). Correction for range restriction: an expanded typology. The Journal of Applied Psychology, 85(1), 112–118. DOI: 10.1037/0021-9010.85.1.112 10740961

[35] Sánchez-Casas, R. M., García-Albea, J. E., & Davis, C. W. (1992). Bilingual lexical processing: Exploring the cognate/non-cognate distinction. European Journal of Cognitive Psychology, 4(4), 293–310. DOI: 10.1080/09541449208406189

[36] Schepens, J., Dijkstra, T., & Grootjen, F. (2012). Distributions of cognates in Europe as based on Levenshtein distance. Bilingualism: Language and Cognition, 15(1), 157–166. DOI: 10.1017/S1366728910000623

[37] Schulpen, B., Dijkstra, T., Schriefers, H. J., & Hasper, M. (2003). Recognition of interlingual homophones in bilingual auditory word recognition. Journal of Experimental Psychology: Human Perception and Performance, 29(6), 1155–1178. DOI: 10.1037/0096-1523.29.6.1155 14640836

[38] Schwartz, A. I., & Kroll, J. F. (2006). Bilingual lexical activation in sentence context. Journal of Memory and Language, 55(2), 197–212. DOI: 10.1016/j.jml.2006.03.004

[39] Schwartz, A. I., Kroll, J. F., & Diaz, M. (2007). Reading words in Spanish and English: Mapping orthography to phonology in two languages. Language and Cognitive Processes, 22(1), 106–129. DOI: 10.1080/01690960500463920

[40] Smits, E., Martensen, H., Dijkstra, T., & Sandra, D. (2006). Naming interlingual homographs: Variable competition and the role of the decision system. Bilingualism: Language and Cognition, 9(3), 281–297. DOI: 10.1017/S136672890600263X

[41] Thorndike, R. L. (1949). Personnel selection: Test and measurement techniques. Oxford, UK: Wiley.

[42] Titone, D., Libben, M., Mercier, J., Whitford, V., & Pivneva, I. (2011). Bilingual lexical access during L1 sentence reading: The effects of L2 knowledge, semantic constraint, and L1–L2 intermixing. Journal of Experimental Psychology: Learning, Memory, and Cognition, 37(6), 1412–1431. DOI: 10.1037/a0024492 21767061

[43] Tokowicz, N., Kroll, J. F., De Groot, A. M. B., & Van Hell, J. G. (2002). Number-of-translation norms for Dutch–English translation pairs: A new tool for examining language production. Behavior Research Methods, Instruments, & Computers, 34(3), 435–451. DOI: 10.3758/BF03195472 12395560

[44] Van Assche, E., Drieghe, D., Duyck, W., Welvaert, M., & Hartsuiker, R. J. (2011). The influence of semantic constraints on bilingual word recognition during sentence reading. Journal of Memory and Language, 64(1), 88–107. DOI: 10.1016/j.jml.2010.08.006

[45] Van Assche, E., Duyck, W., Hartsuiker, R. J., & Diependaele, K. (2009). Does bilingualism change native-language reading? Cognate effects in a sentence context. Psychological Science, 20(8), 923–927. DOI: 10.1111/j.1467-9280.2009.02389.x 19549082

[46] Van Casteren, M., & Davis, M. (2007). Match: A program to assist in matching the conditions of factorial experiments. Behavior Research Methods, 39(4), 973–978. DOI: 10.3758/BF03192992 18183914

[47] Van Hell, J. G., & De Groot, A. M. B. (1998). Conceptual representation in bilingual memory: Effects of concreteness and cognate status in word association. Bilingualism: Language and Cognition, 1(3), 193–211. DOI: 10.1017/S1366728998000352

[48] Van Hell, J. G., & De Groot, A. M. B. (2008). Sentence context modulates visual word recognition and translation in bilinguals. Acta Psychologica, 128(3), 431–451. DOI: 10.1016/j.actpsy.2008.03.010 18486085

[49] Van Hell, J. G., & Dijkstra, T. (2002). Foreign language knowledge can influence native language performance in exclusively native contexts. Psychonomic Bulletin & Review, 9(4), 780–789. DOI: 10.3758/BF03196335 12613683

[50] Van Heuven, W. J. B., Mandera, P., Keuleers, E., & Brysbaert, M. (2014). SUBTLEX-UK: A new and improved word frequency database for British English. The Quarterly Journal of Experimental Psychology, 67(6), 1176–1190. DOI: 10.1080/17470218.2013.850521 24417251

[51] Van Heuven, W. J. B., Schriefers, H. J., Dijkstra, T., & Hagoort, P. (2008). Language conflict in the bilingual brain. Cerebral Cortex, 18(11), 2706–2716. DOI: 10.1093/cercor/bhn030 18424776 PMC2567421

[52] Von Studnitz, R. E., & Green, D. W. (2002). Interlingual homograph interference in German–English bilinguals: Its modulation and locus of control. Bilingualism: Language and Cognition, 5(1), 1–23. DOI: 10.1017/S1366728902000111

[53] Wikipedia. (2014). Lijst van valse vrienden. Retrieved from https://nl.wikipedia.org/wiki/Lijst_van_valse_vrienden#Engels

[54] Yarkoni, T., Balota, D., & Yap, M. J. (2008). Moving beyond Coltheart’s *N*: A new measure of orthographic similarity. Psychonomic Bulletin & Review, 15(5), 971–979. DOI: 10.3758/PBR.15.5.971 18926991

[55] Yudes, C., Macizo, P., & Bajo, T. (2010). Cognate effects in bilingual language comprehension tasks. NeuroReport, 21(7), 507–512. DOI: 10.1097/WNR.0b013e328338b9e1 20375706

